# Outdoor and indoor monitoring of livestock-associated *Culicoides* spp. to assess vector-free periods and disease risks

**DOI:** 10.1186/s12917-016-0710-z

**Published:** 2016-06-04

**Authors:** Katharina Brugger, Josef Köfer, Franz Rubel

**Affiliations:** Institute for Veterinary Public Health, University of Veterinary Medicine Vienna, Veterinaerplatz 1, Vienna, 1210 Austria

**Keywords:** African horse sickness, Basic reproduction number, Bluetongue, Climate change, *Culicoides*-borne diseases, Seasonally vector-free period

## Abstract

**Background:**

Within the last few decades *Culicoides* spp. (Diptera: Ceratopogonidae) emerged Europe-wide as a major vector for epizootic viral diseases e.g. caused by Bluetongue (BT) or Schmallenberg virus. In accordance with the EU regulation 1266/2007, veterinary authorities are requested to determine vector-free periods for loosing trade and movement restrictions of susceptible livestock. Additionally, the widely used basic reproduction number $\mathcal {R}_{0}$ is optionally applied for risk assessment of vector-borne diseases. Values of $\mathcal {R}_{0}<1$ indicate periods with no disease transmission risk. For the determination of vector-free period and $\mathcal {R}_{0}$ a continuously operating daily *Culicoides* spp. monitoring in Vienna (Austria) was established. It covered the period 2009–2013 and depicts the seasonal vector abundance indoor and outdoor. Future BT and African horse sickness (AHS) outbreak risks were estimated by projecting $\mathcal {R}_{0}$ to climate change scenarios. Therefore, temperature-dependent vector parameters were applied.

**Results:**

The vector-free period lasted about 100 days inside stables, while less than five *Culicoides* were trapped outdoors on 150 days per season, i.e. winter half year. Additionally, the potential outbreak risk was assessed for BT and AHS. For BT, a basic reproduction number of $\mathcal {R}_{0}>1$ was found each year between June and August. The periods without transmission risk, i.e. $\mathcal {R}_{0}<1$, were notably higher (200 days). Contrary, values of $\mathcal {R}_{0}<1$ were estimated for AHS during the whole period. Finally, the basic reproduction numbers were projected to the future by using temperature forecasts for the period 2014–2100. While the mean summer peak values for BT increase from of $\mathcal {R}_{0}=2.3$ to $\mathcal {R}_{0}=3.4$ until 2100 (1.1/100 years), no risk for AHS was estimated even under climate warming assumptions.

**Conclusions:**

Restrictions to trade and movement are always associated with an economic impact during epidemic diseases. To minimize these impacts, risk assessments based on the vector-free period or the basic reproduction number $\mathcal {R}_{0}$ can essentially support veterinary authorities to improve protection and control measurements.

## Background

Within the last few decades *Culicoides* spp. (Diptera: Ceratopogonidae) emerged Europe-wide to be a major vector for epizootic viral diseases caused by Bluetongue (BT) virus [1] or Schmallenberg virus [2]. Moreover, these diseases are always associated with a large economic impact, since mainly farm animals are affected. In the case of BT virus, this includes not only production losses due to reduced milk yield, decreased fertility and abortions, but also costs for vaccination programs or trade restrictions [3–6]. In the case of another virus disease at risk of introduction in Europe, the African horse sickness (AHS), an epidemic can have devastating effects on the horse industry due to high mortality rates and strict movement controls as seen e.g. in Spain in the late 1980’s [7].

Recent BT-epidemics in Europe lead to progressive filling of knowledge gaps in life cycles, distributions, habitats and ecology of most European *Culicoides* species [8, 9]. As for instance, to get a first impression on the occurrence and geographical distribution of *Culicoides* spp., the European Union (EU) initialized a large-scale entomological surveillance program in accordance with EU regulation 1266/2007. As a result of this entomological survey, *Culicoides* of the Obsoletus complex, known as important BT virus serotype 8 vectors, are found to be the most widely distributed livestock-associated species in the warm temperate fully humid climate in Central Europe [1, 10]. In the Mediterranean climate, however, *Culicoides imicola* is the predominate vector for BT virus serotypes 1, 2, 4, 6 and 9 and AHS [11].

The knowledge of the seasonal population dynamics is essential for a wide range of applications, e.g. timed stabling as an efficient host protection [12], determination of the seasonally vector-free period, or risk assessments for veterinary authorities to establish protection and control measures. The vector-free period is one criterion for the BT seasonally free period to enable safe movements of susceptible livestock. Within the last years the basic reproduction number $\mathcal {R}_{0}$ has been increasingly used as an epidemiological key parameter for estimating the risk of a disease outbreak [13–16]. Among others, $\mathcal {R}_{0}$ considers the temperature dependent extrinsic incubation period as well as the vector density, or more exactly the vector-to-host ratio. As the vector density is rather unknown, a *Culicoides* monitoring was established at the campus of the University of Veterinary Medicine, Vienna (Austria). Here, a time series with daily outdoor *Culicoides* spp. catches covering the period 2009–2013 is presented. Additionally, a time series with daily indoor *Culicoides* spp. catches for 2009–2011 is provided. So far, only long-term studies of weekly (e.g. [17]) or biweekly [18] outdoor catches were published. Also collections on a daily base are rare and can be found only for short periods like one month [19] or one year [20]. On the other hand, studies inside a stable are very seldom and short [21].

Here, the application of these time series to assess the vector-free period and the risk of a potential disease outbreak by means of BT and AHS is demonstrated. Finally, these outbreak risks were projected to the future by applying temperature-dependent vector parameters [16] to climate change scenarios.

## Methods

### *Culicoides* monitoring

**Fig. 1:**
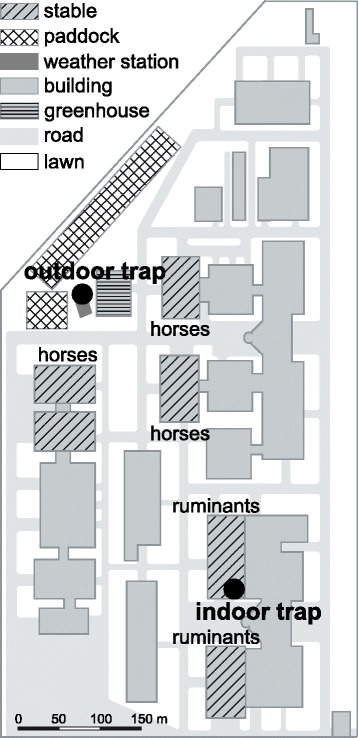
Schematic map of the clinic and hospital area at the University of Veterinary Medicine Vienna. The two *Culicoides* light traps (*circles*), the weather station *Wien - Donaufeld* as well as stables and paddocks for ruminants and horses are marked

The study was carried out at the campus of the University of Veterinary Medicine Vienna (Austria) in the area of the clinics and the animal hospital (Fig. 1). The university is located in the north-western suburb of Vienna, adjacent to housing complexes, detached houses with gardens and agriculture arable fields. For the *Culicoides* monitoring an ultra-violet suction trap from the ARC-Onderstepoort Veterinary Institute (Pretoria, Republic of South Africa) [22] was selected. As this trap type is the OIE gold standard for *Culicoides* spp. trapping, it is also used within the large-scale entomological surveillance program of the EU, e.g. in Austria [23] and Switzerland [17]. We retrofitted the trap with a collection bottle rotator (model 1512, John W. Hock Company, FL, USA) with eight beakers for segregated collections at daily interval (from morning 8:00 a.m. to the next morning 8:00 a.m. local time). To avoid wetting by rain and snow the trap was covered by a plastic canopy. The trap was hung outdoors next to the horse stables and paddocks at a height of 1.5 m above ground [24]. The surrounding vegetation is characterised by deciduous trees, shrubbery and lawn. The trap was operated daily from January 2009 to December 2013.

Additionally, a second trap was placed inside a cattle stable, a tie-stall system with a maximum up to 16 cattle and seldom also alpacas or lamas. To avoid any damages by farm animals and any disturbance of the daily routine, the trap was placed on a window board in 2 m height. Thereto we reconstruct the trap into a metal frame of 80 × 40 × 30 cm. Instead of one beaker, a circular shelve with 12 smaller beakers, which changed every 2 hours, later every 12 h, was installed. This trap was active from March 2009 to November 2011.

For species evaluation the catches were separated first in *Culicoides* spp. and other insects (bycatches) under a stereomicroscope. Afterwards the *Culicoides* spp. were determined by the characteristic pattern and coloration of the wings according to the common identification keys [24–26]. Because this study focuses on the population dynamics, it was mainly differentiated between the two complexes Obsoletus and Pulicaris, respectively. These two complexes are well known vectors for BT virus serotype 8 [27] and supposed vectors for AHS virus [28]. The catches were not separated in male and female nor in physiological stage.

### Vector-free period and disease risk

Generally, by determining a bluetongue seasonally free period veterinary authorities can loosen movement and trade restrictions on susceptible livestock. In accordance to the EU regulation 1266/2007, this comprises no evidence of BT transmission and a seasonally vector-free period. The latter is defined by the absence of *Culicoides* species below a given threshold, currently the total absence of *Culicoides imicola* specimens and less than five parous *Culicoides* per trap. Applied to the Austrian situation, the first point is not appropriate, as so far no *Culicoides imicola* have been detected neither in the Austrian official monitoring [23] nor in the European species distribution [10]. The *Culicoides* complexes Obsoletus and Pulicaris are occurring nationwide in Austria.

To quantify the risk of a potential infectious disease outbreak the basic reproduction number $\mathcal {R}_{0}$ is used. Based on the fundamental equations of Ross and Macdonald [29], $\mathcal {R}_{0}$ has been recently applied for various vector-borne diseases, such as Bluetongue [14, 16], Rift Valley Fever [30] or Usutu virus [13, 31]. It is defined as the number of secondary cases caused by a single infected individual (index case) in an entirely susceptible population. Therefore $\mathcal {R}_{0}$ can be interpreted as a threshold for an outbreak ($\mathcal {R}_{0}>1$) or fade out ($\mathcal {R}_{0}<1$) of a disease [32].

Here the basic reproduction number is applied for BT and AHS, respectively. The equation reads as follows

$${}\mathcal{R}_{0} = \sqrt{\frac{k(T)^{2} \, p_{M} \, \gamma_{M}(T)}{m_{M}(T) \, [\gamma_{M}(T) + m_{M}(T)]} \frac{N_{M}}{(\sum_{i} N_{i})^{2}} \sum_{i} \frac{p_{i} N_{i}}{\alpha_{i}+\nu_{i}} } $$

The basic reproduction number $\mathcal {R}_{0}$ is a function of (partly temperature dependent) vector- or virus-specific parameters (Table 1) and the vector and host densities (Table 2). Most rates were determined in laboratory or field studies, while the host densities N _*i*_ were calculated from the mean numbers of animals at the university campus divided by 1 km ^2^, the campus area. The indices i represent the involved hosts, which are cattle N _*C*_ and small ruminants N _*S*_ for BT, and equids N _*E*_ for AHS [14, 16, 33]. The vector density, here the density of the midges N _*M*_, was estimated by assuming that trap catches reflect 1 % of the local vector population [14]. For estimating $\mathcal {R}_{0}$, daily mean temperature data were provided from the automatic weather station *Wien-Donaufeld* at the university campus. This station is an official weather station (synop. nr. 11090) of the Austrian meteorological network located at geographical coordinates 16.431° E/48.257° N, 161 m above sea level.

**Table 1:** Parameters and parameter functions as applied for calculation of the basic reproduction numbers $\mathcal {R}_{0}$ for African horse sickness (AHS) and Bluetongue (BT). Rates are given in day ^−1^

Parameter	Symbol	Value/Function	Reference
Vector biting rate	k(T)	0.00017 *T* (*T*−3.70) (41.87−*T*)^1/2.71^	[51]
Virus reproduction rate in vector	*γ*_*M*_(T)	0.017 (*T*−12.6)	[52]
Vector mortality rate	*m*_*M*_(T)	0.0089 *e* *x* *p*(0.155 *T*)	[53]
Transmission probability vector to host	*p*_*M*_ for AHS	0.780	[54]
	*p*_*M*_ for BT	1.000	[14]
Transmission probability host to vector	*p*_*E*_ for AHS	0.040	[54]
	*p*_*C*_ = *p*_*S*_ for BT	0.050	[14]
Removal rate of hosts	*α*_*E*_ for AHS	0.125	[55]
	*α*_*C*_ for BT	0.055	[56]
	*α*_*S*_ for BT	0.125	[14]
Fraction dying due to infection	*ν*_*E*_ for AHS	0.800	[55]
	*ν*_*C*_ = *ν*_*S*_ for BT	0.000	[14]

**Table 2:** Vector and host densities used in this study. Units in individuals/km ^2^

Parameter	Symbol	Value/Function
Density of midges	N _*M*_	monitoring 2009–2013
		projection 2014–2100:
		*e* *x* *p* (0.00056+0.1922 *T*) 100
Density of equids	N _*E*_	100
Density of cattle	N _*C*_	25
Density of small ruminants	N _*S*_	25

### Projection to climate change scenarios

For investigating a possible $\mathcal {R}_{0}$ trend under various climate change scenarios, the monthly vector population dynamics was estimated by a Poisson regression model [16]. Therefore, the vector density *N*_*M*_ was derived as

$$ N_{M} = exp \; (0.00056+0.1922 \; T) \cdot 100 $$

depending solely on the monthly mean temperature T. The latter was taken from the TYN SC 2.0 dataset provided by the Tyndall Centre for Climate Change Research [34]. For the period 2014–2100, time series of monthly temperatures were extracted and processed as described by Brugger and Rubel [13]. The worst-case scenario corresponds to an average temperature increase of about 6.1 °C/100 years, while the best-case scenario corresponds to an increase of only 2.6 °C/100 years.

All analyses were conducted using the open-source statistical computing environment R [35]. The source code as well as the digital *Culicoides* spp. time series are provided at the website http://epidemic-modeling.vetmeduni.ac.at.

## Results

Within the observational period 2009–2013 a total of 38,053 *Culicoides* spp. were sampled with the outdoor trap. In detail midges of the Obsoletus complex were the most abundant species (82.8 %) followed by the Pulicaris complex (14.8 %). Inside the stable a total of 36,474 *Culicoides* spp. were caught within almost 3 years (2009–2011). Similar to outside, Obsoletus complex (77.2 %) was the dominating species, followed by the Pulicaris complex (8.5 %). Mainly due to the repair and service of the traps or electrical power outage, there are data gaps for the outdoor trap (7 days, 0.4 %) and for the indoor trap (162 days, 14.8 %, mainly in the winter months). To ensure a continuous time series for calculating $\mathcal {R}_{0}$, missing values of the outdoor trap were replaced by 0 (rainy days) or weekly means.

**Fig. 2:**
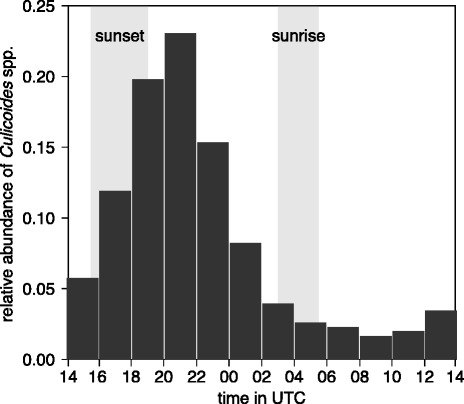
Indoor flight activity of *Culicoides* spp. observed during European summer time (March–October). Time is given in UTC, periods of sunrise and sunset are depicted in grey bars. Period: 2009–2010

To gain daily flight activity periods, we captured *Culicoides* spp. with the indoor trap daily in two hours intervals from beginning of April to end of October in 2009 and 2010, the so-called European summer time. As depicted in Fig. 2, the majority of midges were active from dusk to the first part of the night with a peak between 20–22 UTC (23.0 %). Contrary to the findings in The Netherlands [36] and Germany [37], no secondary peak in the morning hours was observed,because the light trap on the window board was illuminated by the morning sun.

**Fig. 3:**
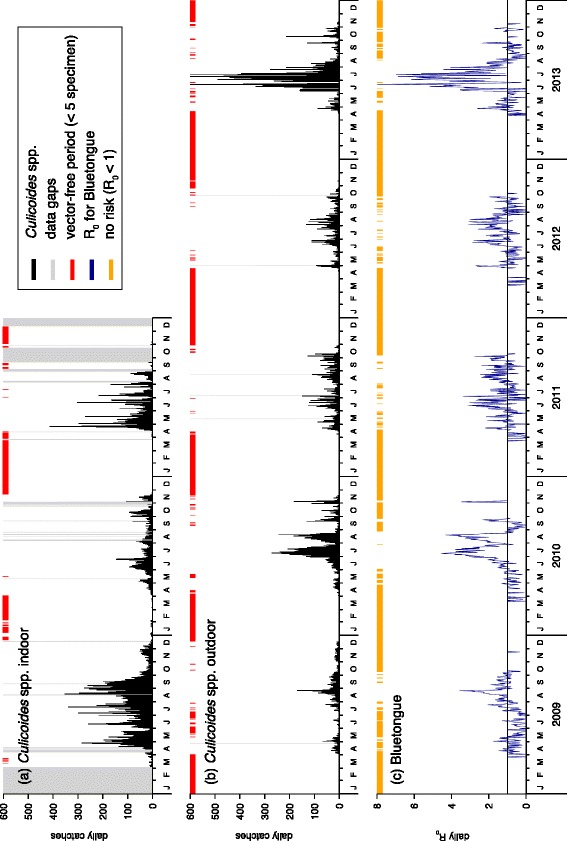
Time series of the daily *Culicoides* spp. catches and the basic reproduction number $\mathcal {R}_{0}$ *Culicoides* were trapped (**a**) indoor and (**b**) outdoor, respectively. The vector-free period, i.e. days with less than five trapped specimen, are given in red. **c** The basic reproduction number are taken as indicator for a potential Bluetongue disease outbreak at the Vetmeduni Vienna (Austria). Days with no risk, i.e. $\mathcal {R}_{0}<0$, are given in orange. Period: 2009–2013

The annual cycles of the *Culicoides* spp. catches indoor (Fig. 3a) and outdoor (Fig. 3b) show very low numbers of midges caught during the winter months and a maximum in the summer (June, July, August). Inter-annual amplitudes were very similar. However, the late spring 2010 and the early summer 2013 were characterised by high precipitation, followed by very warm periods. These weather conditions resulted in notable higher *Culicoides* catches in July/August 2010 and June/July 2013 compared the other years.

According to the EU regulation 1266/2007, both time-series of daily *Culicoides* spp. catches were considered to determine the vector-free period of no virus transmission risk. Less than five specimens were trapped inside the stable on approximately 100 days per season, i.e. winter half year (Fig. 3a), while outdoors the vector-free period lasted for around 150 days (Fig. 3b). These 150 days are of the same order of magnitude as the vector-free period notified to the EU by neighbouring countries [38]. As summarized in Table 3, vector-free periods of about 120 days (range 72–152 days) per season were reported by the Czech Republic, Hungary, Italy, Slovakia, and Switzerland. Thus, the vector-free period can roughly be determined between December and March of the following year. In detail, vector activity decreased markedly in the autumn months and occurred mainly outdoors to a standstill after six consecutive freezing days (T _*min*_<0 °C). In spring, the beginning of the vector season was observed when seven consecutive days with mean daily temperature greater than 10 °C were reached.

**Table 3:** Lengths and dates of the seasonally vector-free periods for Austria and neighbouring countries [38]

	2008/2009		2009/10		2010/11		2011/12		2012/13	
Vector-free period estimated for Austria										
indoor	–	na-05.03.	60	04.02.–04.04.	149	20.11.-17.04.	–		–	
outdoor	–	na-04.04.	113	17.12.–08.04.	143	18.11.-09.04.	180	29.10.–25.04.	162	11.11.–21.04.
$\mathcal {R}_{0}$	–	na-11.04.	204	08.10.–29.04.	167	06.11.-20.04.	204	06.10.–26.04	201	05.10.–23.04.
Vector-free period as officially notified to the EU										
Czech Republic	135	01.12.-14.04.	140	11.12.–29.04.	152	29.11.-29.04.	–		–	
Hungary	–	24.12.-na	133	22.12.–03.05.	-		–		–	
Italy	–	08.12.-na	77	14.12.–28.02.	72	19.12.-28.02.	85	19.12.–13.03.	76	15.12.–28.02.
Slovakia	–		138	17.12.–03.05.	144	06.12.-28.04.	–		–	
Switzerland	138	04.12.–20.04.	131	18.12.–27.04.	121	01.12.-31.03.	–		–	

Units in days, not available dates are indicated with na

Considering not only the vector population dynamics, but also the virus transmission cycle, the basic reproduction number $\mathcal {R}_{0}$ emerges as crucial parameter to assess the risk of a potential disease outbreak in Vienna at the university campus. For BT, a basic reproduction number above one, indicating a risk for a major disease outbreak, was generally found between July and August (Fig. 3c). Maximum daily values were equal $\mathcal {R}_{0}=8$, which can be interpreted as eight secondary cases caused by one primary case at the beginning of an epidemic. A period of $\mathcal {R}_{0}<1$ was estimated for about 200 days per season. Contrary, for AHS values of $\mathcal {R}_{0}<1$ were estimated for the whole period except three days with extraordinary high numbers of *Culicoides* spp. in 2013. Thus, a major AHS outbreak in Vienna is extreme unlikely.

**Fig. 4:**
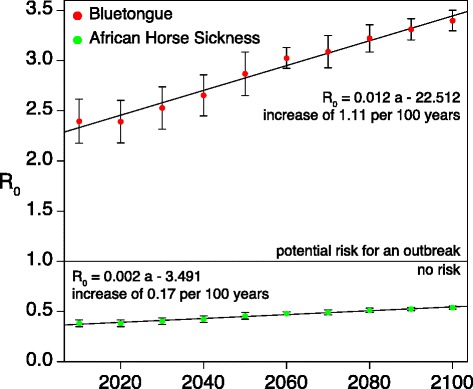
Projection of future basic reproduction numbers $\mathcal {R}_{0}$ for Bluetongue and African horse sickness in Vienna, Austria. The peak values of the summer months (June, July, August) of each year were averaged over 10 years

In the course of rising temperatures as a consequence of climate change, an increasing linear trend was calculated for the basic reproduction numbers. For BT, the values of $\mathcal {R}_{0}$ were estimated to increase between 0.59/100 years (best-case scenario) and 1.11/100 years (worst-case scenario). Projected $\mathcal {R}_{0}$ values for AHS remain below 1, although a small increasing trend of 0.17/100 years was calculated for the worst-case scenario (Fig. 4).

## Discussion

This paper presents, to our knowledge, a unique time series of continuously operating daily *Culicoides* monitoring for five years outdoors and three years indoors. Although light traps were blamed in recent years for not being representative to reflect *Culicoides* spp. activity in a host [39, 40], they are the only suitable method for long-term monitoring. Amongst others, such time series of high temporal resolution are needed to reliably simulate the seasonal abundance of a vector population. This includes not only the most frequently applied statistical models [16, 41], but also dynamical models that are not yet available for *Culicoides* spp. vectors. Subsequently, vector models may be used as an integral part of epidemic models to simulate the spatio-temporal spread of a vector-borne disease [42, 43].

During BT outbreaks, vector monitorings were directly used to define vector-free periods in accordance with the EU regulation 1266/2007. In contrast to the risk assessment based on $\mathcal {R}_{0}$, the absence of vectors is defined with no outbreak risk. For this alternative approach a threshold of five parous *Culicoides* specimens per trap is recommended. Here, five *Culicoides* specimens were assumed instead of five parous females. This rather strict interpretation of the EU regulation was applied to avoid uncertainties concerning the determination of parous females [44, 45] and to consider the possibility of transovarial virus transmission [46]. Based on our Culicoides monitoring, a vector-free period of approximately 150 days per season can be defined. This is one quarter shorter than the period without transmission risk, i.e. $\mathcal {R}_{0}<1$ (200 days per year). Thus, risk assessments based on $\mathcal {R}_{0}$ should be preferred, because it additionally considers the virus transmission cycle and allows a projection to the future by using temperature predictions from climate models. The latter results in a slightly increasing risk for BT outbreaks corresponding to increasing $\mathcal {R}_{0}$ values until the end of the century. In accordance with expert opinions [47] we estimated no risk for an AHS outbreak in Vienna neither for today nor for the future years. As the study site is located in the warmest region of the country, the results can be considered as a worst-case scenario for Austria. Nevertheless, in the course of the recent detection of BT virus serotype 4, eight additional *Culicoides* traps were installed in December 2015 throughout Austria to confirm the vector-free period. Further improvements may be achieved by operating different trap types for negative control [48]. However, veterinary authorities currently not realised this option for financial reasons.

## Conclusion

Outbreak numbers caused by *Culicoides* spp. transmitted viruses in Europe are increasing steadily over the last two decades. Recent examples include the re-emerging of BT virus serotype 8 in France [49] or the northward-spreading of BT virus serotype 4 along the Balkan Peninsula [50]. In case of an outbreak, veterinary authorities have to react promptly and rapidly to control the spread of the epidemic and then to contain the associated economic impact. Here, we applied two methods for setting the scale during which no virus transmission is expected. Thereby, the vector-free period can be interpreted as maximum timeframe and the risk estimation based on $\mathcal {R}_{0}$ as minimum. Risk assessments based on the basic reproduction number $\mathcal {R}_{0}$ should be preferred as they can essentially support veterinary authorities to improve protection and control measures. Further, the critical vaccination proportion can be directly derived from $\mathcal {R}_{0}$.

## Abbreviations

AHS, African horse sickness; BT, bluetongue; EU, European union.
